# Association between obesity and remission in rheumatoid arthritis patients treated with disease-modifying anti-rheumatic drugs

**DOI:** 10.1038/s41598-020-75673-7

**Published:** 2020-10-29

**Authors:** Ahmad Y. Abuhelwa, Ashley M. Hopkins, Michael J. Sorich, Susanna Proudman, David J. R. Foster, Michael D. Wiese

**Affiliations:** 1 Department of Clinical Pharmacology, College of Medicine and Public Health, Flinders University, Bedford Park, SA 5042 Australia; 2 Clinical and Health Sciences, University of South Australia, Adelaide, SA 5000 Australia; 3 Royal Adelaide Hospital, Adelaide, SA 5000 Australia; 4 Discipline of Medicine, University of Adelaide, Adelaide, SA 5000 Australia

**Keywords:** Prognostic markers, Rheumatology, Rheumatic diseases, Rheumatoid arthritis

## Abstract

The aim of this study was to investigate the association between body-mass index (BMI) and remission in RA patients receiving conventional synthetic (cs-) or the biological Disease-Modifying Antirheumatic Drug (DMARD), tocilizumab. Individual participant data (IPD) were pooled from five trials investigating tocilizumab and/or csDMARDs therapy (primarily methotrexate) for RA. Time to first remission was recorded according to the Simplified Disease Activity Index (SDAI) and Clinical Disease Activity Index (CDAI). BMI was classified according to WHO definitions. Associations between baseline BMI and remission were assessed by Cox-proportional hazard analysis. IPD were available from 5428 patients treated with tocilizumab ± csDMARDs (n = 4098) or csDMARDs alone (n = 1330). Of these, 1839 (33.9%) had normal BMI, 1780 (32.8%) overweight, 1652 (30.4%) obese and 157 (2.9%) were underweight. Obesity, compared to normal BMI, was associated with less frequent remission using SDAI (adjusted HR 0.80 [95% CI 0.70–0.92]) and CDAI (adjusted HR 0.77 [0.68–0.87]). As continuous variable, increased BMI was associated with less frequent SDAI (P = 0.001) and CDAI (P = 0.001) defined remission. No heterogeneity in identified associations was observed between studies (P = 0.08) or treatments (P = 0.22). Obesity was negatively associated with RA disease remission regardless of RA therapy, suggesting that baseline BMI should be considered as a stratification factor in future RA trials.

## Introduction

Rheumatoid arthritis (RA) is a chronic inflammatory autoimmune disease characterised by tender and swollen joints that leads to irreversible joint deformity, disability and diminished quality of life^1^. Conventional synthetic disease modifying anti-rheumatic drugs [csDMARDs—e.g. methotrexate (MTX), hydroxychloroquine, leflunomide and sulfasalazine] and biological DMARDs [bDMARDs—e.g. the interleukin-6 receptor blocker, tocilizumab (TCZ)] are the current backbone of treatment that aims to control inflammation and prevent irreversible outcomes of uncontrolled RA^1^.

Adipose tissue is considered an active participant in modulating physiological and pathological processes associated with inflammation and immunity, and excess body weight, measured by body mass index (BMI), has been suggested to be associated with various autoimmune/inflammatory conditions^2^. Adipose tissue produces and secretes a wide range of proinflammatory factors, including adipokines such as leptin, as well as cytokines such as tissue necrosis factor-α (TNF-α), interleukin-1β (IL-1β), IL-6 and monocyte chemotactic protein-1^3,4^. Obesity, characterised by excess accumulation of adipose tissue, is associated with elevated levels of proinflammatory adipokines^3^.

Various epidemiological studies have reported obesity to be a risk factor for RA development^5–7^. Preliminary research also indicates that patients with newly diagnosed RA who are obese or overweight may be less likely to achieve good response and low disease activity^8,9^. Obesity has also been found to reduce the probability of response to anti-tissue necrosis factor (anti-TFN) agents^10,11^. Conversely, some studies indicate elevated BMI may be associated with less radiographic joint damage^12,13^. Therefore, the association between BMI with RA outcomes requires further clarification. Further, there is no clear indication as to whether or not the association with BMI is the same in patients using csDMARDs versus bDMARDs, which have fundamental pharmacological differences. The aim of this study was to use large, high-quality individual-participant data (IPD) collected within randomised control trials (RCTs) to investigate the association between BMI and remission in RA patients receiving csDMARDs and/or the bDMARD, TCZ.

## Methods

### Patient population

IPD from the Hoffmann-La Roche sponsored phase III clinical trials LITHE (clinicaltrials.gov identifier NCT00106535, registered 28 March 2005), AMBITION (NCT00109408, registered 28 April 2005), TOWARD (NCT00106574, registered 28 March 2005), FUNCTION (NCT01007435, registered 20 August 2012), and SUMMACTA (NCT01194414, registered 3 September 2010) were utilized in this pooled analysis. The de-identified individual participant data analysed during the current study were accessed via Vivli, Inc, in line with Roche’s public data transparency policy. The authors are independent researchers with no affiliation to Vivli or Roche. All studies were conducted in accordance with the International Conference on Harmonization guidelines for Good Clinical Practice and the ethical principles of the Declaration of Helsinki. Written informed consent was obtained from all patients prior to enrolment. The secondary analysis of de-identified IPD, in the current study, was exempted from review by the Southern Adelaide Local Health Network, Office for Research and Ethics as it was classified as minimal risk research.

LITHE included RA patients randomly assigned (1:1:1) to TCZ (4 or 8 mg/kg) plus MTX or MTX alone^14^. AMBITION included RA patients randomized 3:3:1 to TCZ (8 mg/kg), MTX (7.5 mg/week) or placebo for 8 weeks followed by TCZ (8 mg/kg)^15^. TOWARD randomised patients 2:1 to either TCZ (8 mg/kg) or placebo, with both groups receiving concomitant csDMARD therapy^16^. FUNCTION included MTX-naïve patients with early progressive RA randomized 1:1:1:1 to TCZ (4 or 8 mg/kg) plus MTX, TCZ (8 mg/kg) or MTX^17^. SUMMACTA included RA patients randomised 1:1 to TCZ-subcutaneous (TCZ-SC) 162 mg weekly or TCZ-intravenous (TCZ-IV) 8 mg/kg every four weeks for 24 weeks in combination with csDMARDs. To assess the long term safety and efficacy of TCZ in an extension study of SUMMACTA, patients who received TCZ-SC in the first 24 weeks were randomised 11:1 to receive TCZ-SC or TCZ-IV and patients receiving TCZ-IV were randomized 2:1 to receive TCZ-IV or TCZ-SC^18^. Data was collected up to week 97 in the SUMMACTA extension study^18^.

All studies included adult patients (age ≥ 18 years) diagnosed with moderate to severe RA for ≥ 3 (AMBITION) or ≥ 6 months (all other studies) according to American College of Rheumatology (ACR) classification criteria. Active RA was defined by swollen joint count (SJC) ≥ 6 (66 joint count), tender joint count (TJC) ≥ 8 (68 joint count) and C-reactive protein (CRP) ≥ 1 mg/dl or erythrocyte sedimentation rate (ESR) ≥ 28 mm/h. FUNCTION included early progressive RA patients defined according to the 28-joint Disease Activity Score using the erythrocyte sedimentation rate (DAS28-ESR) over 3.2, with SJC ≥ 4, TJC ≥ 6, ESR ≥ 28 mm/h or CRP ≥ 1 mg/dl and positive rheumatoid factor or anti-cyclic citrullinated peptide antibodies.

### Predictors and outcomes

The primary outcome was time to first RA disease remission according to Simplified Disease Activity Index (SDAI ≤ 3.3) as per the 2010 ACR/EULAR criteria^19^. Time to first RA disease remission according to Clinical Disease Activity Index (CDAI ≤ 2.8) was the secondary outcome^19^. Patients were censored at the last known date of follow up or at the recorded date of death if they had not achieved remission.

The primary assessed covariate was pre-treatment (i.e. value at baseline) body mass index (BMI). BMI was calculated as total body weight (kg) divided by the square of body height (m^2^). BMI was categorised according to standard WHO definitions (underweight < 18.5, normal 18.5–25.0, overweight 25.0–30.0 and obese > 30.0 kg/m^2^). Data on pre-treatment SDAI, CDAI, age, sex, race, RA disease duration, number of previous DMARDs, corticosteroid use, presence of hypertension, coronary artery disease or diabetes mellitus was available.

The individual components of the disease activity measures (TJC, SJC, CRP, physician and patient assessments of disease activity) were also evaluated to highlight which components of disease activity measure are the main drivers of the reduced remission rate in obese participants. Remission criteria for individual disease activity measures were based on the Boolean criteria proposed by Felson et al.^19^, whereby remission was defined for TJC as ≤ 1, SJC ≤ 1, and CRP ≤ 1 mm/h. Although not included in Felson et al., physician assessment of disease activity < 10% was classified as remission.

### Statistical analysis

Cox proportional hazard analysis was used to assess the association between pre-treatment BMI and remission. Results were reported as hazard ratios (HR) with 95% confidence intervals (95% CI). BMI was initially modelled as a continuous variable. Potential non-linear associations were evaluated using restricted cubic splines and visual checks. Model fit was assessed according to the Akaike information criterion and c-statistic. Analyses were conducted with a focus on facilitating clinical use and interpretability. Thus, the association between BMI categories and remission was also assessed. Statistical significance was set at a threshold of P = 0.05 (likelihood ratio test). Univariable and adjusted analyses were conducted—adjusted analyses were conducted to assess the independence of associations from other known prognostic factors. All analyses were stratified by study and treatment arm. Heterogeneity of remission likelihood according to patient BMI and received treatment or enrolled study was assessed using a treatment-by-biomarker interaction term in the Cox proportional regression model. Kaplan–Meier analysis was used for plotting and estimating remission probabilities. All analyses were conducted using R version 3.4.3.

### Ethics declaration

These studies were conducted in accordance with the International Conference on Harmonization guidelines for Good Clinical Practice and the ethical principles of the Declaration of Helsinki. Written informed consent was obtained from all patients prior to enrolment. The secondary analysis of de-identified IPD, in the current study, was exempted from review by the Southern Adelaide Local Health Network, Office for Research and Ethics as it was classified as minimal risk research.

## Results

### Patient population

The pooled analysis cohort consisted of 5502 patients, of whom 4126 (75%) were treated with TCZ ± csDMARDs and 1376 (25%) with csDMARDs alone (primarily methotrexate). A summary of patient characteristics by study cohort and BMI category is provided in Table 1 and Supplementary file: Tables S1, respectively. BMI was not available for 65 (1.2%) patients and SDAI was missing for 9 (0.2%), leaving 5428 available for the SDAI-based remission analysis. Of these, 1839 (33.9%) had a normal BMI, 1780 (32.8%) were overweight, 1652 (30.4%) were obese and 157 (2.9%) were underweight. No significant heterogeneity in BMI distribution was observed between trials (P = 0.226).

**Table 1:** Baseline characteristics of RA patients in each study cohort.

Variable	Total	Lithe	Ambition	Toward	Function	Summacta	P
Total	5502 (100%)	1190 (21.6%)	673 (12.2%)	1220 (22.2%)	1157 (21%)	1262 (22.9%)	
**Actual ARM**							< 0.001
MTX	284 (5.2%)	0 (0%)	284 (42.2%)	0 (0%)	0 (0%)	0 (0%)	
Placebo (8 weeks) then TCZ 8 mg/kg	101 (1.8%)	0 (0%)	101 (15%)	0 (0%)	0 (0%)	0 (0%)	
Placebo + DMARDs	415 (7.5%)	0 (0%)	0 (0%)	415 (34%)	0 (0%)	0 (0%)	
Placebo + MTX	677 (12.3%)	392 (32.9%)	0 (0%)	0 (0%)	285 (24.6%)	0 (0%)	
TCZ-IV to TCZ-SC	186 (3.4%)	0 (0%)	0 (0%)	0 (0%)	0 (0%)	186 (14.7%)	
TCZ-SC to TCZ-IV	48 (0.9%)	0 (0%)	0 (0%)	0 (0%)	0 (0%)	48 (3.8%)	
TCZ 162 mg SC qw + DMARD	583 (10.6%)	0 (0%)	0 (0%)	0 (0%)	0 (0%)	583 (46.2%)	
TCZ 4 mg/kg + MTX	689 (12.5%)	399 (33.5%)	0 (0%)	0 (0%)	290 (25.1%)	0 (0%)	
TCZ 8 mg/kg	288 (5.2%)	0 (0%)	288 (42.8%)	0 (0%)	0 (0%)	0 (0%)	
TCZ 8 mg/kg + DMARDs	805 (14.6%)	0 (0%)	0 (0%)	805 (66%)	0 (0%)	0 (0%)	
TCZ 8 mg/kg + MTX	689 (12.5%)	399 (33.5%)	0 (0%)	0 (0%)	290 (25.1%)	0 (0%)	
TCZ 8 mg/kg + Placebo	292 (5.3%)	0 (0%)	0 (0%)	0 (0%)	292 (25.2%)	0 (0%)	
TCZ 8 mg/kg IV q4w + DMARD	445 (8.1%)	0 (0%)	0 (0%)	0 (0%)	0 (0%)	445 (35.3%)	
**Treatment type**							< 0.001
csDMARDs	1376 (25%)	392 (32.9%)	284 (42.2%)	415 (34%)	285 (24.6%)	0 (0%)	
TCZ ± csDMARDs	4126 (75%)	798 (67.1%)	389 (57.8%)	805 (66%)	872 (75.4%)	1262 (100%)	
Age (years)	53 [44–61]	53 [44–61]	51 [42–59]	54 [46–62]	51 [41–60]	54 [45–62]	< 0.001
**Age group**							0.006
26–35	500 (9.1%)	106 (8.9%)	69 (10.3%)	85 (7%)	133 (11.5%)	107 (8.5%)	
36–45	920 (16.7%)	201 (16.9%)	126 (18.7%)	183 (15%)	208 (18%)	202 (16%)	
46–55	1660 (30.2%)	376 (31.6%)	209 (31.1%)	363 (29.8%)	330 (28.5%)	382 (30.3%)	
56–65	1498 (27.2%)	319 (26.8%)	163 (24.2%)	354 (29%)	306 (26.4%)	356 (28.2%)	
≥ 65	924 (16.8%)	188 (15.8%)	106 (15.8%)	235 (19.3%)	180 (15.6%)	215 (17%)	
**Weight at baseline (kg)**	71 [60.4–84.4]	70 [60–82.7]	70.5 [60.5–83.1]	70.95 [60.5–84.8]	71.8 [61–85]	72 [60.12–85.45]	0.291
Missing	47 (0.9%)	41 (3.4%)	4 (0.6%)	2 (0.2%)	0 (0%)	0 (0%)	
**Height at baseline (cm)**	162 [156.5–168]	161.5 [156–168]	163 [157–170]	162 [157–168]	163 [157–170]	162 [156–168]	< 0.001
Missing	24 (0.4%)	8 (0.7%)	4 (0.6%)	3 (0.2%)	5 (0.4%)	4 (0.3%)	
**BMI at baseline**	26.8 [23.36–31.2]	26.83 [23.23–30.87]	26.4 [23.5–31]	26.86 [23.31–31.2]	26.75 [23.28–30.83]	27.15 [23.6–31.9]	0.220
Missing	65 (1.2%)	49 (4.1%)	4 (0.6%)	3 (0.2%)	5 (0.4%)	4 (0.3%)	
**BMI categories at baseline**							0.225
Normal	1844 (33.9%)	399 (35%)	226 (33.8%)	408 (33.5%)	397 (34.5%)	414 (32.9%)	
Obese	1654 (30.4%)	338 (29.6%)	191 (28.6%)	365 (30%)	337 (29.3%)	423 (33.6%)	
Overweight	1782 (32.8%)	380 (33.3%)	236 (35.3%)	401 (32.9%)	381 (33.1%)	384 (30.5%)	
Underweight	157 (2.9%)	24 (2.1%)	16 (2.4%)	43 (3.5%)	37 (3.2%)	37 (2.9%)	
Missing	65 (1.2%)	49 (4.1%)	4 (0.6%)	3 (0.2%)	5 (0.4%)	4 (0.3%)	
**Sex**							0.013
F	4474 (81.3%)	989 (83.1%)	538 (79.9%)	1002 (82.1%)	904 (78.1%)	1041 (82.5%)	
M	1028 (18.7%)	201 (16.9%)	135 (20.1%)	218 (17.9%)	253 (21.9%)	221 (17.5%)	
**Race**							< 0.001
Asian	407 (7.4%)	68 (5.7%)	48 (7.1%)	118 (9.7%)	89 (7.7%)	84 (6.7%)	
Black	250 (4.5%)	60 (5%)	34 (5.1%)	63 (5.2%)	34 (2.9%)	59 (4.7%)	
Other	776 (14.1%)	224 (18.8%)	93 (13.8%)	160 (13.1%)	145 (12.5%)	154 (12.2%)	
White	4069 (74%)	838 (70.4%)	498 (74%)	879 (72%)	889 (76.8%)	965 (76.5%)	
**RA Disease duration in years**	3.9 [0.84–10.8]	7.33 [3.14–13.54]	3.14 [0.64–9.44]	6.98 [2.84–14.42]	0.24 [0.1–0.63]	5.95 [2.33–12.24]	< 0.001
Missing	12 (0.2%)	0 (0%)	0 (0%)	1 (0.1%)	0 (0%)	11 (0.9%)	
No. of previous DMARDS	1 [0–2]	2 [1–3]	1 [0–2]	1 [0–2]	0 [0–0]	2 [2, 3]	< 0.001
**No of previous DMARDS**							< 0.001
1	1197 (21.8%)	294 (24.7%)	162 (24.1%)	318 (26.1%)	219 (18.9%)	204 (16.2%)	
2 or more	2563 (46.6%)	716 (60.2%)	222 (33%)	550 (45.1%)	19 (1.6%)	1056 (83.7%)	
None	1742 (31.7%)	180 (15.1%)	289 (42.9%)	352 (28.9%)	919 (79.4%)	2 (0.2%)	
**Anti-citrullinated protein antibodies**							1.000
Negative	519 (9.4%)	0 (0%)	0 (0%)	0 (0%)	193 (16.7%)	326 (26.2%)	
Positive	1874 (34.1%)	0 (0%)	0 (0%)	0 (0%)	954 (82.5%)	920 (73.8%)	
Missing	3109 (56.5%)	1190 (100%)	673 (42.9%)	1220 (100%)	10 (0.9%)	16 (1.3%)	
**Diabetes**							< 0.001
Y	402 (7.3%)	48 (4%)	45 (6.7%)	114 (9.3%)	91 (7.9%)	104 (8.2%)	
N	5100 (92.7%)	1142 (96%)	628 (93.3%)	1106 (90.7%)	1066 (92.1%)	1158 (91.8%)	
**Hypertension**							0.022
Y	1740 (31.6%)	378 (31.8%)	189 (28.1%)	382 (31.3%)	350 (30.3%)	441 (34.9%)	
N	3762 (68.4%)	812 (68.2%)	484 (71.9%)	838 (68.7%)	807 (69.7%)	821 (65.1%)	
**Coronary artery disorder**							0.497
Y	162 (2.9%)	35 (2.9%)	15 (2.2%)	32 (2.6%)	35 (3%)	45 (3.6%)	
N	5340 (97.1%)	1155 (97.1%)	658 (97.8%)	1188 (97.4%)	1122 (97%)	1217 (96.4%)	
**Corticosteroids**							< 0.001
N	2260 (41.1%)	359 (30.2%)	320 (47.5%)	507 (41.6%)	591 (51.1%)	483 (38.3%)	
Y	3242 (58.9%)	831 (69.8%)	353 (52.5%)	713 (58.4%)	566 (48.9%)	779 (61.7%)	
**CRP (mg/L)**	1.49 [0.64–3.2]	1.42 [0.62–3]	1.8 [0.74–3.88]	1.49 [0.64–3.19]	1.45 [0.57–3.21]	1.47 [0.68–3]	< 0.001
Missing	54 (1%)	8 (0.7%)	10 (1.5%)	17 (1.4%)	7 (0.6%)	12 (1%)	
**ESR (mm/h)**	43 [30–64]	40 [29–59]	44 [30–63]	42 [30–63]	45 [30–69.25]	45 [32–67]	< 0.001
Missing	44 (0.8%)	11 (0.9%)	1 (0.1%)	16 (1.3%)	1 (0.1%)	15 (1.2%)	
**Swollen joint 28 count**	11 [8–16]	11 [7–15]	12 [8–17]	12 [8–17]	10 [7–16]	10 [7–15]	< 0.001
Missing	10 (0.2%)	1 (0.1%)	2 (0.3%)	7 (0.6%)	0 (0%)	0 (0%)	
**Tender joint 28 count**	15 [10–21]	14 [10–19]	16 [11–22]	15 [10–21]	15 [10–22]	15 [10–21]	< 0.001
Missing	10 (0.2%)	1 (0.1%)	2 (0.3%)	7 (0.6%)	0 (0%)	0 (0%)	
**Provider GH**	64 [51–75]	64 [52–74]	65 [53–76]	65 [52–75]	65 [51–76]	62 [50–75]	0.043
Missing	14 (0.3%)	9 (0.8%)	1 (0.1%)	3 (0.2%)	0 (0%)	1 (0.1%)	
**Patient GH**	68 [51–82]	65 [47.75–79]	68 [50–81]	69 [51–83]	68 [52–83]	70 [52–84]	< 0.001
Missing	28 (0.5%)	10 (0.8%)	5 (0.7%)	11 (0.9%)	1 (0.1%)	1 (0.1%)	
**(Event = remission, Censored: not)**							< 0.001
Censored	4019 (73.2%)	684 (57.5%)	595 (88.4%)	1119 (92.3%)	706 (61%)	915 (72.6%)	
Event	1473 (26.8%)	505 (42.5%)	78 (11.6%)	93 (7.7%)	451 (39%)	346 (27.4%)	
Missing	10 (0.2%)	1 (0.1%)	0 (0%)	8 (0.7%)	0 (0%)	1 (0.1%)	
**SDAI score**	41.79 [32.39–52.02]	39.87 [32.31–49.99]	44.27 [35.66–53.22]	42.88 [33.14–52.94]	41.87 [31.39–53.6]	40.92 [31.55–51.67]	< 0.001
Missing	97 (1.8%)	27 (2.3%)	17 (2.5%)	31 (2.5%)	8 (0.7%)	14 (1.1%)	
**SDAI score categorized**							0.006
Low activity	9 (0.2%)	2 (0.2%)	0 (0%)	1 (0.1%)	5 (0.4%)	1 (0.1%)	
Moderate activity	588 (10.9%)	124 (10.7%)	47 (7.2%)	126 (10.6%)	146 (12.7%)	145 (11.6%)	
High activity	4808 (89%)	1037 (89.2%)	609 (92.8%)	1062 (89.3%)	998 (86.9%)	1102 (88.3%)	
Missing	97 (1.8%)	27 (2.3%)	17 (2.5%)	31 (2.5%)	8 (0.7%)	14 (1.1%)	
**CDAI score**	39.4 [30.5–49.2]	38.1 [30.4–47.1]	41.45 [33.12–50.27	40.05 [31–50.12]	39.45 [29.8–50.3]	38.5 [29.4–48.8]	< 0.001
Missing	45 (0.8%)	19 (1.6%)	7 (1%)	16 (1.3%)	1 (0.1%)	2 (0.2%)	
**CDAI score categorized**							0.012
Low activity	10 (0.2%)	2 (0.2%)	0 (0%)	1 (0.1%)	6 (0.5%)	1 (0.1%)	
Moderate activity	372 (6.8%)	79 (6.7%)	28 (4.2%)	82 (6.8%)	84 (7.3%)	99 (7.9%)	
High activity	5075 (93%)	1090 (93.1%)	638 (95.8%)	1121 (93.1%)	1066 (92.2%)	1160 (92.1%)	
Missing	45 (0.8%)	19 (1.6%)	7 (1%)	16 (1.3%)	1 (0.1%)	2 (0.2%)	

Data are median (interquartile range) or number of patients (%).

*TCZ* tocilizumab, *MTX* methotrexate, *SDAI* simplified disease activity index, *CDAI* clinical disease activity index, *BMI* body mass index, *DMARD* disease-modifying antirheumatic drugs, *CRP* C-reactive protein, *ESR* erythrocyte sedimentation rate, *GH* global health.

For the CDAI-based remission analysis, CDAI was missing for 2 (0.1%) patients, leaving 5435 patients for analysis, of whom 1842 (33.9%) had a normal BMI, 1782 (32.8%) were overweight, 1654 (30.4%) were obese and 157 (2.9%) were underweight. No significant heterogeneity in BMI distribution was observed between trials (P = 0.226).

Median follow-up was 260 weeks in LITHE, 24 weeks in AMBITION, 24 weeks in TOWARD, 52 weeks in FUNCTION and 97 weeks in SUMMACTA.

### Association between BMI and remission outcomes

**Figure 1:**
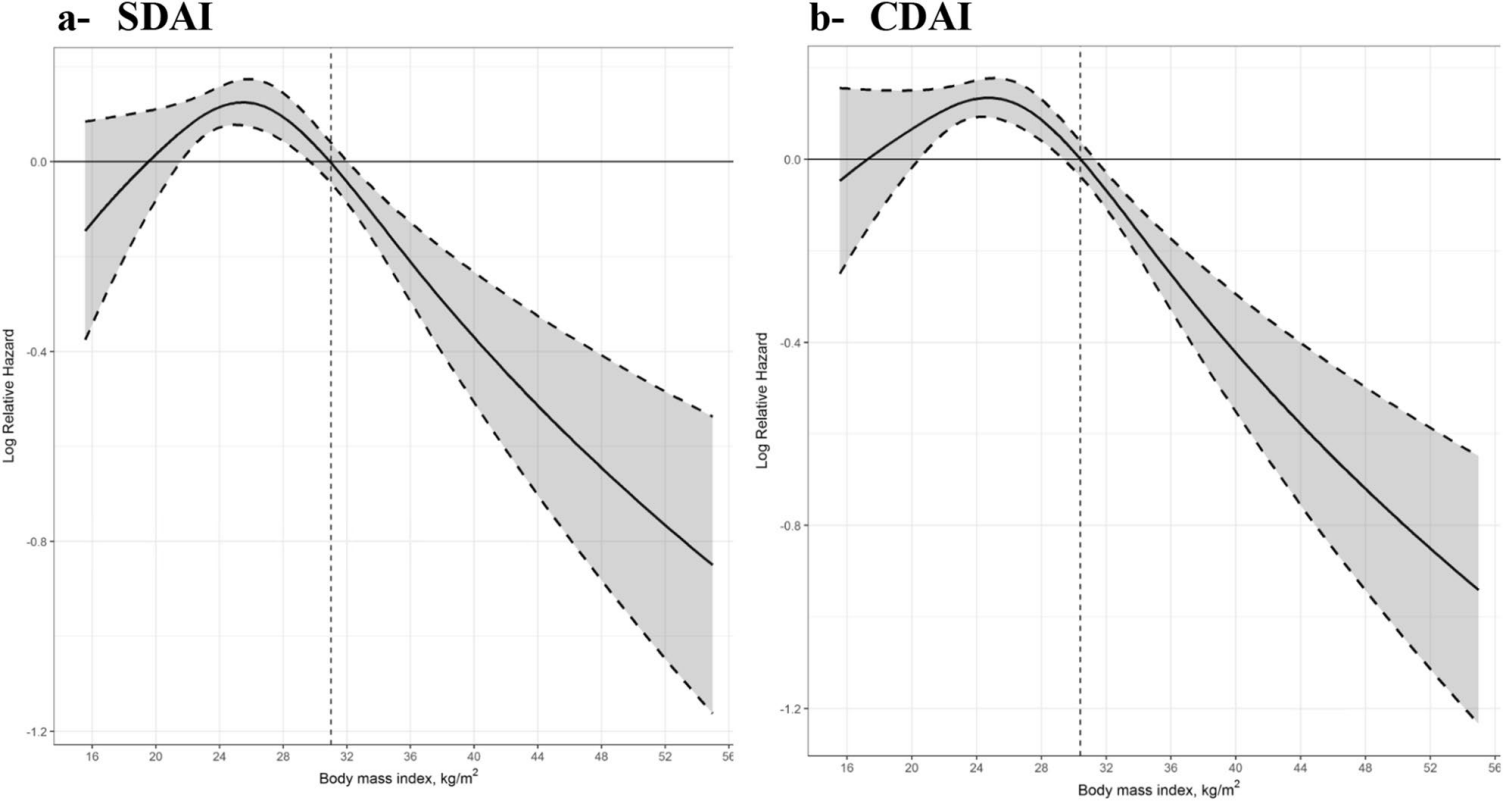
Log-relative hazard curves describing the continuous association between BMI and remission using (**A**) simplified disease activity index (SDAI) and (**B**) clinical disease activity index (CDAI) remission. Log-relative hazard curves solid line represents the average log hazard, shaded area is the 95% confidence interval, and the vertical dashed line represents a BMI cut point of 30 kg/m^2^.

The continuous association between BMI and remission was best described using a restricted cubic spline with three knots. Increased BMI was associated with less frequent SDAI (P = 0.001) and CDAI (P = 0.001) defined remission. Figure 1 describes the continuous association between BMI and remission and shows that remission rate became progressively worse when BMI increased to over 30.

**Figure 2:**
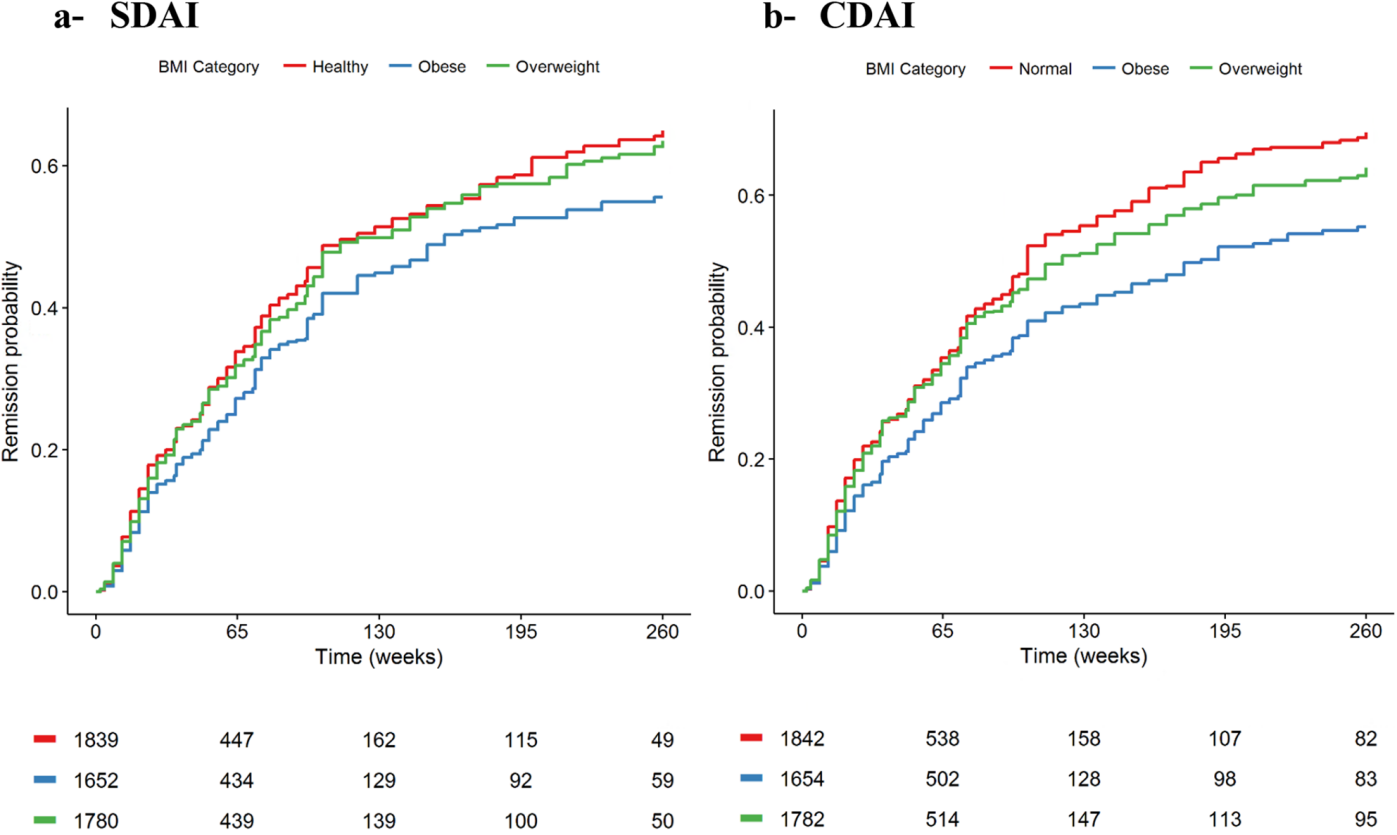
Kaplan–Meier estimates of proportion of rheumatoid arthritis patients achieving remission at least once by BMI category in the pooled cohort using (**A**) simplified disease activity index (SDAI) and (**B**) clinical disease activity index (CDAI) remission. The numbers underneath Kaplan–Meier plots indicate the absolute number of patients at risk by time.

BMI category was significantly associated with different remission likelihood on univariable (Table 2, P = 0.001) and adjusted (Table 3, P = 0.01) analyses. Using univariable analysis, obesity, compared to normal BMI was associated with less frequent remission using SDAI (HR 0.78 [95% CI 0.68–0.89]) and CDAI (HR 0.72 [95% CI 0.63–0.81]) based criteria. Using adjusted analysis, obesity was also associated with less frequent remission using SDAI (HR 0.80 [95% CI 0.70–0.92]) and CDAI (HR 0.77 [95% CI 0.68–0.87]) based criteria. Figure 2 presents Kaplan Meier estimates of remission likelihood according to BMI category.

**Table 2:** Univariable analysis of the association between BMI and remission in the pooled cohort.

Pooled cohort	SDAI-remission			CDAI-remission		
	Events/patients	HR [95% CI]	P	Events/patients	HR [95% CI]	P
**BMI category**			0.001			0.001
Normal	538/1839	1		677/1842	1	
Overweight	494/1780	0.95 [0.84–1.07]		600/1782	0.92 [0.83–1.03]	
Obese	401/1652	0.78 [0.68–0.89]		449/1654	0.72 [0.63–0.81]	
Underweight	32/157	0.72 [0.51–1.03]		47/157	0.8 [0.60–1.08]	

*CI* confidence interval, *HR* hazard ratio, *BMI* body mass index (kg/m^2^), *SDAI* simplified disease activity index, *CDAI* clinical disease activity index.

**Table 3:** Adjusted analysis of the association between BMI and remission in the pooled cohort.

Pooled cohort	SDAI-remission			CDAI-remission		
	Events/patients	HR [95% CI]	P	Events/patients	HR [95% CI]	P
**BMI category**			0.01			0.001
Normal	527/1802	1		669/1825	1	
Overweight	488/1748	0.95 [0.84–1.08]		596/1761	0.95 [0.85–1.07]	
Obese	399/1630	0.80 [0.70–0.92]		447/1642	0.77 [0.68–0.87]	
Underweight	s31/152	0.83 [0.57–1.19]		46/155	0.85 [0.63–1.15]	

Adjustment variables: age, race, sex, RA disease duration, presence of coronary artery diseases, hypertension, diabetes, corticosteroid use, baseline SDAI score, baseline CDAI score, and number of previous DMARDs.

*CI* confidence interval, *HR* hazard ratio, *BMI* body mass index (kg/m^2^), *SDAI* simplified disease activity index, *CDAI* clinical disease activity index.

Further, the analysis suggests that underweight patients tend to have a lower remission rate compared to normal BMI (Table 3, Supplementary file: Fig. S2). However, this association was not statistically significant using SDAI (HR 0.83 [0.57–1.19]) and CDAI based criteria (HR 0.85 [0.63–1.15]).

The identified association between obesity and remission was consistent between studies (Supplementary file: Table S2, interaction P = 0.08) and treatment arms (Supplementary file: Table S3, interaction P = 0.22), suggesting that this association is irrespective of the type of RA therapy.

The analysis of the individual components of the disease activity measures revealed that TJC and the physician assessment of disease activity were the two primary drivers for reduced remission rate in participants who were obese compared to those with a normal BMI. Kaplan–Meier plots and a table summary of the Cox-proportional hazard analysis of the individual components of disease activity measures by BMI category are provided in the supplementary material (Supplementary file: Table S4 and Fig. S1).

## Discussion

This analysis that included large, multiple, independent cohorts of RA patients receiving TCZ and/or csDMARDs (primarily methortrexate) showed that patients who were obese were less likely to achieve remission compared to those with a normal BMI, whereas outcomes were no different in those who were overweight or underweight compared to those who had a normal BMI. These results were consistent with analyses modelling BMI as a continuous variable which showed that outcome became progressively worse when BMI increased over 30 kg/m^2^.

The strength of the present analysis is the large number of RA patients from well-conducted RCTs and the ability to adjust the analysis for potential confounders, which increases the reliability and validity of the findings. The poorer outcomes in obese patients were independent of age, race, sex, RA disease duration, presence of coronary artery disease, hypertension, diabetes, corticosteroid use, number of previous DMARDs and baseline disease activity.

The association between obesity and remission is consistent with previous studies that investigated the association between BMI and response to a number of anti-TNF-α agents, including weight-adjusted infliximab treatment^10,11^ and treatment combining infliximab with either MTX or MTX, sulfasalazine and prednisolone^20^. Most recently, Schafer et al. demonstrated that obesity had a negative impact on improvement in disease activity (measured via DAS28-ESR) in patients with RA who had received csDMARDs or biologic DMARDs, including 1173 patients receiving tocilizumab^21^. Levitsky et al. have also shown that obesity was associated with worse outcomes in patients randomized to combination csDMARDs; however, no significant differences were identified for patients randomized to infliximab plus MTX^9^, which may have been due to the small number of patients included in this arm (n = 128). In contrast to Levitsky et al. and Schafer et al. studies^9,21^, the analysis presented herein included IPD pooled from 5 RCTs and 5502 patients, of whom 4212 patients received treatment containing TCZ, and RA remission was defined using ACR/EULAR criteria^19^ which are more stringent in defining remission than the DAS28 used in the Levitsky et al. and Schafer et al. studies^22^.

Unlike the findings of this analysis where no significant association between overweight and remission were found, Sandberg et al. reported that being overweight decreased the chance of achieving good disease control in RA patient receiving DMARDs (86% methotrexate)^23^. However, this was an observational study that included a relatively small number of patients (n = 495) and obese and overweight patients were grouped together as patients that had a BMI > 25 kg/m^2^, whereas the present analysis was able to differentiate between the associations with BMI in these patient groups.

The association between underweight BMI category and remission was not the primary focus of this analysis as there were only 157 [2.9%] underweight patients available for analysis. A much larger sample size of underweight participants would be needed to draw conclusions with regards to the association between underweight BMI category and remission.

Although studies included in this analysis were not designed to elucidate biologic mechanisms, adipose tissue has been shown to modulate physiological and pathological processes associated with inflammation and immunity including RA^24^. Adipose tissue secretes a number of proinflammatory cytokines, including TNF-α and IL-6^25^. Thus, obese patients are expected to have higher levels of inflammatory mediators compared to those with a normal BMI, which may worsen RA disease activity and outcomes^25^, or it may simply be that greater amounts of TCZ are required to overcome this higher baseline level of cytokines. However, adipose tissue also secretes anti-inflammatory substances such as adiponectin, which may explain the paradox reported in some publications suggesting high BMI may protect against, or at least not hasten joint destruction^20,26,27^.

A limitation of this analysis is that there might be other confounders that were not available in the clinical trial data and hence were not included in the analysis. Potential confounders that were not available include concurrent fibromyalgia, osteoarthritis and educational status. Another limitation of this study is that the outcome was defined by time to first remission. This measure does not represent the overall time that a patient is in remission, and other outcomes such as time in remission or sustained remission may be a better indicator of long-term joint damage^28^. However, since some of the studies included in the analysis had a relatively short follow-up, the use of time in remission as an outcome measure is likely to be limited. Furthermore, whether the association between BMI and outcomes is consistent over time and extends to joint damage remains unanswered and radiographic outcomes were not available in the data. Except for in the SUMMACTA study where TCZ was administered via subcutaneous injection, TCZ was administered intravenously in the remaining studies. Given that TCZ is now commonly used subcutaneously, future research will have a role in determining if this has any impact on the identified relationship. Finally, whether weight loss will increase responsiveness to DMARDs requires further investigation.

## Conclusion

The findings of our analysis suggest that obese patients are less likely to achieve RA remission regardless of the type of DMARD used. These results endorse the notion that adipose tissue may be involved in the pathophysiology of RA and raises the possibility that baseline BMI should be considered as a stratification factor in future RA therapy trials. Studies investigating whether weight loss improves the responsiveness to DMARDs in RA patients need to be further investigated.

## Supplementary information

Supplementary Information.

## Data Availability

This publication is based on research using de-identified individual participant data from data contributor Hoffmann-La Roche that has been made available through Vivli, Inc. Vivli has not contributed to or approved, and is not in any way responsible for, the content of this publication.

## Abbreviations

ACR: American College of Rheumatology
bDMARDs: Biological disease-modifying anti-rheumatic drugs
BMI: Body mass index
CDAI: Clinical disease activity index
CRP: C-reactive protein
csDMARDs: Conventional-synthetic disease-modifying anti-rheumatic drugs
DAS28-ESR: Disease Activity Score using the erythrocyte sedimentation rate
DMARDs: Disease-modifying anti-rheumatic drugs
ESR: Erythrocyte sedimentation rate
HR: Hazard ratio
IPD: Individual-participant data
IV: Intravenous
MTX: Methotrexate
RA: Rheumatoid arthritis
RCT: Randomised control trials
SC: Subcutaneous
SDAI: Simplified disease activity index
SJC: Swollen joint count
TCZ: Tocilizumab
TJC: Tender joint count

## References

[1] Smolen JS, Aletaha D, McInnes IB (2016). Rheumatoid arthritis. Lancet.

[2] Versini M, Jeandel P-Y, Rosenthal E, Shoenfeld Y (2019). Mosaic of Autoimmunity.

[3] Trayhurn, P., Wood, I. Signalling role of adipose tissue: adipokines and inflammation in obesity. Portland Press Ltd. (2005). 10.1042/BST0331078 16246049

[4] Touyz, R. M. Endothelial cell IL-8, a new target for adiponectin: implications in vascular protection. Am Heart Assoc. (2005). 10.1161/01.RES.0000196745.09234.36 16339493

[5] Feng J (2016). Body mass index and risk of rheumatoid arthritis: A meta-analysis of observational studies. Medicine (Baltimore).

[6] Lu B (2014). Being overweight or obese and risk of developing rheumatoid arthritis among women: A prospective cohort study. Ann. Rheum. Dis..

[7] Crowson CS, Matteson EL, Davis JM, Gabriel SE (2013). Contribution of obesity to the rise in incidence of rheumatoid arthritis. Arthritis Care Res. (Hoboken).

[8] Liu Y, Hazlewood GS, Kaplan GG, Eksteen B, Barnabe C (2017). Impact of obesity on remission and disease activity in rheumatoid arthritis: A systematic review and meta-analysis. Arthritis Care Res. (Hoboken).

[9] Levitsky A (2017). Obesity is a strong predictor of worse clinical outcomes and treatment responses in early rheumatoid arthritis: Results from the SWEFOT trial. RMD Open.

[10] Gremese E (2013). Obesity and reduction of the response rate to anti-tumor necrosis factor α in rheumatoid arthritis: An approach to a personalized medicine. Arthritis Care Res..

[11] Klaasen R, Wijbrandts CA, Gerlag DM, Tak PP (2011). Body mass index and clinical response to infliximab in rheumatoid arthritis. Arthritis. Rheum..

[12] Baker JF (2014). Greater body mass independently predicts less radiographic progression on X-ray and MRI over 1–2 years. Ann. Rheum. Dis..

[13] Hashimoto J (2009). A combination of biochemical markers of cartilage and bone turnover, radiographic damage and body mass index to predict the progression of joint destruction in patients with rheumatoid arthritis treated with disease-modifying anti-rheumatic drugs. Mod. Rheumatol..

[14] Kremer JM (2011). Tocilizumab inhibits structural joint damage in rheumatoid arthritis patients with inadequate responses to methotrexate: Results from the double-blind treatment phase of a randomized placebo-controlled trial of tocilizumab safety and prevention of structural joint damage at 1 year. Arthritis. Rheum..

[15] Jones G (2010). Comparison of tocilizumab monotherapy versus methotrexate monotherapy in patients with moderate to severe rheumatoid arthritis: The AMBITION study. Ann. Rheum. Dis..

[16] Genovese MC (2008). Interleukin-6 receptor inhibition with tocilizumab reduces disease activity in rheumatoid arthritis with inadequate response to disease-modifying antirheumatic drugs: The tocilizumab in combination with traditional disease-modifying antirheumatic drug therapy study. Arthritis. Rheum..

[17] Burmester GR (2016). Tocilizumab in early progressive rheumatoid arthritis: FUNCTION, a randomised controlled trial. Ann. Rheum. Dis..

[18] Burmester GR (2016). Efficacy and safety of subcutaneous tocilizumab versus intravenous tocilizumab in combination with traditional DMARDs in patients with RA at week 97 (SUMMACTA). Ann. Rheum. Dis..

[19] Felson DT (2011). American College of Rheumatology/European League Against Rheumatism provisional definition of remission in rheumatoid arthritis for clinical trials. Arthritis. Rheum..

[20] Heimans L (2013). Association of high body mass index with decreased treatment response to combination therapy in recent-onset rheumatoid arthritis patients. Arthritis. Care Res. (Hoboken).

[21] Schäfer M (2020). Obesity reduces the real-world effectiveness of cytokine-targeted but not cell-targeted disease-modifying agents in rheumatoid arthritis. Rheumatology (Oxford, England).

[22] Rintelen B (2009). Comparison of three rheumatoid arthritis disease activity scores in clinical routine. Scand. J. Rheumatol..

[23] Sandberg ME (2014). Overweight decreases the chance of achieving good response and low disease activity in early rheumatoid arthritis. Ann. Rheum. Dis..

[24] Fantuzzi G (2005). Adipose tissue, adipokines, and inflammation. J. Allergy Clin. Immunol..

[25] Vidal C, Barnetche T, Morel J, Combe B, Daien C (2015). Association of body mass index categories with disease activity and radiographic joint damage in rheumatoid arthritis: A systematic review and metaanalysis. J. Rheumatol..

[26] Westhoff G, Rau R, Zink A (2007). Radiographic joint damage in early rheumatoid arthritis is highly dependent on body mass index. Arthritis Rheum..

[27] Ajeganova S, Andersson ML, Hafström I, Group, B. S (2013). Association of obesity with worse disease severity in rheumatoid arthritis as well as with comorbidities: A long-term followup from disease onset. Arthritis Care Res..

[28] Aletaha D (2009). Rheumatoid arthritis joint progression in sustained remission is determined by disease activity levels preceding the period of radiographic assessment. Arthritis Rheum..

