# Supplementary material for: Association between obesity and remission in rheumatoid arthritis patients treated with disease-modifying anti-rheumatic drugs
Source: Sci Rep. 2020 Oct 29;10:18634. doi: 10.1038/s41598-020-75673-7 (PMC7596471; doi:10.1038/s41598-020-75673-7)
Supplement: Supplementary file 1 — Supplementary Information. [file 41598_2020_75673_MOESM1_ESM.docx]

Supplementary material

# Association between obesity and remission in rheumatoid arthritis patients treated with disease-modifying anti-rheumatic drugs

# Authors

Ahmad Y Abuhelwa^1,2*^, Ashley M Hopkins^1^, Michael J Sorich^1^, Susanna Proudman^3,4^, David J.R Foster^2^, Michael D Wiese^2^.

# Affiliations

1. Department of Clinical Pharmacology, College of Medicine and Public Health, Flinders University, Bedford Park 5042, SA, Australia
2. Clinical and Health Sciences, University of South Australia, Adelaide 5000, SA Australia
3. Royal Adelaide Hospital, Adelaide 5000, SA, Australia
4. Discipline of Medicine, University of Adelaide, Adelaide 5000, SA, Australia

# Corresponding author

Dr Ahmad Y. Abuhelwa, College of Medicine and Public Health, Flinders University, Bedford Park 5042, SA, Australia. Phone: +61413118743 [Ahmad.Abuhelwa@flinders.edu.au](mailto:Ahmad.Abuhelwa@flinders.edu.au)

Table S1. Baseline characteristics of RA patients by BMI category.

| **Variable** | **Total** | **Healthy** | **Obese** | **Overweight** | **Underweight** | **P-value** |
| --- | --- | --- | --- | --- | --- | --- |
| Total | 5502 (100%) | 1844 (33.9%) | 1654 (30.4%) | 1782 (32.8%) | 157 (2.9%) |  |
| Actual ARM     MTX     Placebo (8 weeks) then TCZ 8 mg/kg     Placebo + DMARDs     Placebo + MTX     TCZ-IV to TCZ-SC     TCZ-SC to TCZ-IV     TCZ 162 mg SC qw + DMARD     TCZ 4 mg/kg + MTX     TCZ 8 mg/kg     TCZ 8 mg/kg + DMARDs     TCZ 8 mg/kg + MTX     TCZ 8 mg/kg + Placebo     TCZ 8 mg/kg IV q4w + DMARD | 284 (5.2%) 101 (1.8%) 415 (7.5%) 677 (12.3%) 186 (3.4%) 48 (0.9%) 583 (10.6%) 689 (12.5%) 288 (5.2%) 805 (14.6%) 689 (12.5%) 292 (5.3%) 445 (8.1%) | 96 (5.2%) 32 (1.7%) 139 (7.5%) 217 (11.8%) 70 (3.8%) 15 (0.8%) 196 (10.6%) 239 (13%) 98 (5.3%) 269 (14.6%) 243 (13.2%) 97 (5.3%) 133 (7.2%) | 73 (4.4%) 35 (2.1%) 118 (7.1%) 185 (11.2%) 55 (3.3%) 15 (0.9%) 194 (11.7%) 202 (12.2%) 83 (5%) 247 (14.9%) 196 (11.9%) 92 (5.6%) 159 (9.6%) | 104 (5.8%) 33 (1.9%) 143 (8%) 212 (11.9%) 58 (3.3%) 16 (0.9%) 176 (9.9%) 222 (12.5%) 99 (5.6%) 258 (14.5%) 232 (13%) 95 (5.3%) 134 (7.5%) | 10 (6.4%) 1 (0.6%) 15 (9.6%) 19 (12.1%) 2 (1.3%) 2 (1.3%) 16 (10.2%) 20 (12.7%) 5 (3.2%) 28 (17.8%) 14 (8.9%) 8 (5.1%) 17 (10.8%) | 0.771 |
| Treatment type     csDMARDs     TCZ ± csDMARDs | 1376 (25%) 4126 (75%) | 452 (24.5%) 1392 (75.5%) | 376 (22.7%) 1278 (77.3%) | 459 (25.8%) 1323 (74.2%) | 44 (28%) 113 (72%) | 0.146 |
| Age (years) | 53 [44-61] | 51 [40-59.25] | 54 [47-61] | 54 [45-62] | 48 [33-57] | <0.001 |
| Age group     >=65     26-35     36-45     46-55     56-65 | 924 (16.8%) 500 (9.1%) 920 (16.7%) 1660 (30.2%) 1498 (27.2%) | 318 (17.2%) 250 (13.6%) 339 (18.4%) 511 (27.7%) 426 (23.1%) | 218 (13.2%) 98 (5.9%) 248 (15%) 568 (34.3%) 522 (31.6%) | 334 (18.7%) 121 (6.8%) 295 (16.6%) 527 (29.6%) 505 (28.3%) | 36 (22.9%) 24 (15.3%) 32 (20.4%) 39 (24.8%) 26 (16.6%) | <0.001 |
| Weight at baseline (kg)     Missing | 71 [60.4-84.4] 47 (0.9%) | 59 [54-65] 0 (0%) | 90.5 [82-102.7] 0 (0%) | 72 [66.5-79] 0 (0%) | 45.5 [42-49.1] 0 (0%) | <0.001 |
| Height at baseline (cm)     Missing | 162 [156.5-168] 24 (0.4%) | 162 [157-169] 0 (0%) | 162 [156-167.6] 0 (0%) | 162 [156-170] 0 (0%) | 163 [157-168] 0 (0%) | 0.038 |
| BMI at baseline     Missing | 26.8 [23.36-31.2] 65 (1.2%) | 22.6 [21-23.83] 0 (0%) | 34.09 [31.7-38] 0 (0%) | 27.3 [26.13-28.6] 0 (0%) | 17.6 [16.89-18] 0 (0%) | <0.001 |
| Sex     F     M | 4474 (81.3%) 1028 (18.7%) | 1507 (81.7%) 337 (18.3%) | 1403 (84.8%) 251 (15.2%) | 1369 (76.8%) 413 (23.2%) | 142 (90.4%) 15 (9.6%) | <0.001 |
| Race     Asian     Black     Other     White | 407 (7.4%) 250 (4.5%) 776 (14.1%) 4069 (74%) | 236 (12.8%) 47 (2.5%) 260 (14.1%) 1301 (70.6%) | 26 (1.6%) 125 (7.6%) 199 (12%) 1304 (78.8%) | 90 (5.1%) 73 (4.1%) 285 (16%) 1334 (74.9%) | 53 (33.8%) 4 (2.5%) 21 (13.4%) 79 (50.3%) | <0.001 |
| RA Disease duration in years     Missing | 3.9 [0.84-10.8] 12 (0.2%) | 4.22 [0.93-11.15] 4 (0.2%) | 3.47 [0.86-10.09] 2 (0.1%) | 3.82 [0.78-10.87] 5 (0.3%) | 4.47 [0.92-12.57] 1 (0.6%) | 0.185 |
| No. of previous DMARDS | 1 [0-2] | 1 [0-2] | 1 [0-2] | 1 [0-2] | 1 [0-3] | 0.890 |
| No of previous DMARDS      1     2 or more     None | 1197 (21.8%) 2563 (46.6%) 1742 (31.7%) | 407 (22.1%) 871 (47.2%) 566 (30.7%) | 350 (21.2%) 781 (47.2%) 523 (31.6%) | 401 (22.5%) 831 (46.6%) 550 (30.9%) | 35 (22.3%) 70 (44.6%) 52 (33.1%) | 0.959 |
| Diabetes     Y     N | 402 (7.3%) 5100 (92.7%) | 66 (3.6%) 1778 (96.4%) | 216 (13.1%) 1438 (86.9%) | 113 (6.3%) 1669 (93.7%) | 4 (2.5%) 153 (97.5%) | <0.001 |
| Hypertension     Y     N | 1740 (31.6%) 3762 (68.4%) | 342 (18.5%) 1502 (81.5%) | 805 (48.7%) 849 (51.3%) | 553 (31%) 1229 (69%) | 18 (11.5%) 139 (88.5%) | <0.001 |
| Coronary artery disorder     Y     N | 162 (2.9%) 5340 (97.1%) | 30 (1.6%) 1814 (98.4%) | 65 (3.9%) 1589 (96.1%) | 57 (3.2%) 1725 (96.8%) | 4 (2.5%) 153 (97.5%) | <0.001 |
| Corticosteroids     N     Y | 2260 (41.1%) 3242 (58.9%) | 772 (41.9%) 1072 (58.1%) | 699 (42.3%) 955 (57.7%) | 702 (39.4%) 1080 (60.6%) | 59 (37.6%) 98 (62.4%) | 0.230 |
| CRP (mg/L)     Missing | 1.49 [0.64-3.2] 54 (1%) | 1.6 [0.62-3.59] 24 (1.3%) | 1.41 [0.69-2.7] 12 (0.7%) | 1.43 [0.61-3.1] 13 (0.7%) | 2.34 [0.77-4.41] 3 (1.9%) | <0.001 |
| ESR (mm/h)     Missing | 43 [30-64] 44 (0.8%) | 45 [31-68] 14 (0.8%) | 42 [30-60] 13 (0.8%) | 42 [30-62] 14 (0.8%) | 55.5 [36-81.25] 1 (0.6%) | <0.001 |
| Swollen joint 28 count      Missing | 11 [8-16] 10 (0.2%) | 11 [7-16] 3 (0.2%) | 11 [7-15] 1 (0.1%) | 11 [8-16] 5 (0.3%) | 11 [7-16] 0 (0%) | 0.708 |
| Tender joint 28 count      Missing | 15 [10-21] 10 (0.2%) | 15 [10-20] 3 (0.2%) | 15 [10-22] 1 (0.1%) | 15 [10-21] 5 (0.3%) | 15 [10-21] 0 (0%) | 0.026 |
| Provider GH     Missing | 64 [51-75] 14 (0.3%) | 64 [51-75] 6 (0.3%) | 64 [52-75] 2 (0.1%) | 64 [52-75] 4 (0.2%) | 66 [50-77] 0 (0%) | 0.657 |
| Patient GH     Missing | 68 [51-82] 28 (0.5%) | 66 [49-81] 8 (0.4%) | 69 [51-84] 7 (0.4%) | 69 [51-83] 10 (0.6%) | 70.5 [54-84.5] 1 (0.6%) | 0.014 |
| (Event=remission, Censored: not)     Censored     Event     Missing | 4019 (73.2%) 1473 (26.8%) 10 (0.2%) | 1301 (70.7%) 538 (29.3%) 5 (0.3%) | 1251 (75.7%) 401 (24.3%) 2 (0.1%) | 1286 (72.2%) 494 (27.8%) 2 (0.1%) | 125 (79.6%) 32 (20.4%) 0 (0%) | 0.002 |
| SDAI score     Missing | 41.79 [32.39-52.02] 97 (1.8%) | 41.25 [32.14-51.62] 38 (2.1%) | 41.46 [32.39-51.98] 22 (1.3%) | 42.39 [32.54-52.4] 29 (1.6%) | 42.33 [32.29-53.72] 4 (2.5%) | 0.392 |
| SDAI score Categorized     Low activity     Moderate activity     High activity     Missing | 9 (0.2%) 588 (10.9%) 4808 (89%) 97 (1.8%) | 3 (0.2%) 206 (11.4%) 1597 (88.4%) 38 (2.1%) | 3 (0.2%) 176 (10.8%) 1453 (89%) 22 (1.3%) | 3 (0.2%) 188 (10.7%) 1562 (89.1%) 29 (1.6%) | 0 (0%) 14 (9.2%) 139 (90.8%) 4 (2.5%) | 0.970 |
| CDAI score     Missing | 39.4 [30.5-49.2] 45 (0.8%) | 38.8 [30.3-48.6] 15 (0.8%) | 39.5 [30.5-49.52] 10 (0.6%) | 40 [30.8-49.5] 16 (0.9%) | 39.25 [29.9-49.12] 1 (0.6%) | 0.199 |
| CDAI score Categorized     Low activity     Moderate activity     High activity     Missing | 10 (0.2%) 372 (6.8%) 5075 (93%) 45 (0.8%) | 3 (0.2%) 128 (7%) 1698 (92.8%) 15 (0.8%) | 4 (0.2%) 106 (6.4%) 1534 (93.3%) 10 (0.6%) | 3 (0.2%) 124 (7%) 1639 (92.8%) 16 (0.9%) | 0 (0%) 11 (7.1%) 145 (92.9%) 1 (0.6%) | 0.976 |

Data are median (interquartile range) or number of patients (%). TCZ: tocilizumab; MTX: methotrexate; SDAI: simplified disease activity index; CDAI: clinical disease activity index; BMI: body mass index; DMARD: disease-modifying antirheumatic drugs, CRP: C-reactive protein; ESR: erythrocyte sedimentation rate, GH: global health

Table S2. Adjusted analysis of the association between BMI category and remission by study cohort

|  |  | **SDAI-remission** | | | **CDAI-remission** | | |
| --- | --- | --- | --- | --- | --- | --- | --- |
| **Subgroup** | **Variable** | **Events/ patients** | **HR [95% CI]** | **P** | **Events/ patients** | **HR [95% CI]** | **P** |
| LITHE | BMI category |  |  | 0.033 |  |  | 0.052 |
|  | Normal | 173/392 | 1 |  | 202/393 | 1 |  |
|  | Overweight | 182/369 | 1.14 [0.92‑1.42] |  | 196/373 | 1.01 [0.83‑1.24] |  |
|  | Obese | 123/333 | 0.81 [0.63‑1.03] |  | 136/335 | 0.76 [0.60‑0.96] |  |
|  | Underweight | 7/23 | 0.85 [0.39‑1.87] |  | 11/23 | 1.01 [0.54‑1.89] |  |
| AMBITION | BMI category |  |  | 0.810 |  |  |  |
|  | Normal | 26/223 | 1 |  | 30/224 | 1 | 0.782 |
|  | Overweight | 31/228 | 1.05 [0.61‑1.79] |  | 34/234 | 1.03 [0.62‑1.71] |  |
|  | Obese | 18/185 | 0.80 [0.42‑1.51] |  | 18/188 | 0.77 [0.41‑1.44] |  |
|  | Underweight | 2/16 | 1.30 [0.30‑5.73] |  | 2/16 | 1.23 [0.28‑5.38] |  |
| TOWARD | BMI category |  |  | 0.569 |  |  | 0.346 |
|  | Normal | 32/393 | 1 |  | 45/401 | 1 |  |
|  | Overweight | 23/395 | 0.76 [0.44‑1.31] |  | 36/396 | 0.83 [0.53‑1.30] |  |
|  | Obese | 33/356 | 0.98 [0.58‑1.67] |  | 35/361 | 0.82 [0.50‑1.32] |  |
|  | Underweight | 5/42 | 1.43 [0.55‑3.74] |  | 8/43 | 1.72 [0.80‑3.70] |  |
| FUNCTION | BMI category |  |  |  |  |  |  |
|  | Normal | 163/390 | 1 | 0.420 | 212/397 | 1 | 0.282 |
|  | Overweight | 150/380 | 0.92 [0.73‑1.15] |  | 181/380 | 0.96 [0.78‑1.18] |  |
|  | Obese | 120/337 | 0.82 [0.63‑1.05] |  | 132/337 | 0.82 [0.65‑1.04] |  |
|  | Underweight | 14/37 | 1.12 [0.64‑1.95] |  | 18/37 | 1.23 [0.75‑2.03] |  |
| SUMMACTA | BMI category |  |  | 0.08 |  |  | <0.001 |
|  | Normal | 133/404 | 1 |  | 180/410 | 1 |  |
|  | Overweight | 102/376 | 0.82 [0.63‑1.08] |  | 149/378 | 0.88 [0.70‑1.10] |  |
|  | Obese | 105/419 | 0.73 [0.55‑0.96] |  | 126/421 | 0.71 [0.56‑0.91] |  |
|  | Underweight | 3/34 | 0.30 [0.09‑0.94] |  | 7/37 | 0.31 [0.14‑0.66] |  |

CI=confidence interval, HR=hazard ratio, BMI = body mass index (kg/m^2^).

Adjustment variables: age, race, sex, RA disease duration, presence of coronary artery diseases, hypertension, other vascular disorders (not hypertension), diabetes, corticosteroid use, baseline SDAI score, baseline CDAI score, and number of previous DMARDs.

Table S3. Adjusted analysis of the association between BMI category and remission by treatment cohort.

|  |  | **SDAI-remission** | | | **CDAI-remission** | | |
| --- | --- | --- | --- | --- | --- | --- | --- |
| **Subgroup** | **Variable** | **Events/ patients** | **HR [95% CI]** | **P** | **Events/ patients** | **HR [95% CI]** | **P** |
| TCZ±csDMARDs | BMI category |  |  | 0.088 |  |  | 0.007 |
|  | Normal | 419/1366 | 1 |  | 550/1380 | 1 |  |
|  | Obese | 338/1256 | 0.84 [0.72‑0.98] |  | 380/1267 | 0.79 [0.69‑0.91] |  |
|  | Overweight | 387/1297 | 0.95 [0.82‑1.09] |  | 483/1307 | 0.94 [0.83‑1.06] |  |
|  | Underweight | 23/110 | 0.67 [0.49‑1.16] |  | 38/112 | 0.81 [0.58‑1.13] |  |
| csDMARDs | BMI category |  |  | 0.026 |  |  | 0.053 |
|  | Normal | 108/436 | 1 |  | 119/445 | 1 |  |
|  | Obese | 61/374 | 0.67 [0.48‑0.93] |  | 67/375 | 0.69 [0.50‑0.94] |  |
|  | Overweight | 101/451 | 0.99 [0.75‑1.32] |  | 113/454 | 1.02 [0.78‑1.33] |  |
|  | Underweight | 8/42 | 1.15 [0.55‑2.40] |  | 8/43 | 1.07 [0.51‑2.22] |  |

CI=confidence interval, HR=hazard ratio, BMI = body mass index (kg/m^2^).

Adjustment variables: age, race, sex, RA disease duration, presence of coronary artery diseases, hypertension, other vascular disorders (not hypertension), diabetes, corticosteroid use, baseline SDAI score, baseline CDAI score, and number of previous DMARDs.

Table S4. Adjusted analysis for the association between BMI category and remission for individual disease activity measures

| **Metric** | **Variable** | **HR [95% CI]** | **P** |
| --- | --- | --- | --- |
| Tender joint count ≤ 1 | BMI category |  | 0.003 |
|  | Normal | 1 |  |
|  | Overweight | 0.92 [0.85‑1.01 ] |  |
|  | Obese | 0.85 [0.78‑0.94] |  |
| Swollen joint count ≤ 1 | BMI category |  | 0.240 |
|  | Normal | 1 |  |
|  | Overweight | 1.04 [0.96‑1.13] |  |
|  | Obese | 0.97 [0.88‑1.06] |  |
| Physician assessment of disease activity ≤ 10% | BMI category |  | 0.021 |
|  | Normal | 1 |  |
|  | Overweight | 1.02 [0.94‑1.12] |  |
|  | Obese | 0.90 [0.82‑0.99] |  |
| Patient assessment of disease activity ≤ 10% | BMI category |  |  |
|  | Normal | 1 | 0.262 |
|  | Overweight | 0.97 [0.88‑1.07] |  |
|  | Obese | 0.92 [0.83‑1.02] |  |
| C-reactive protein ≤ 1 mmg/h | BMI category |  |  |
|  | Normal | 1 | 0.056 |
|  | Overweight | 1.09 [1.02‑1.16] |  |
|  | Obese | 1.04 [0.97‑1.12] |  |

CI=confidence interval, HR=hazard ratio, BMI = body mass index (kg/m^2^). Remission was defined as Tender joint count ≤ 1, Swollen joint count ≤ 1, Physician assessment of disease activity ≤ 10%, Patient assessment of disease activity ≤ 10%, C-reactive protein ≤ 1 mm/h.

Adjustment variables: age, race, sex, RA disease duration, presence of coronary artery diseases, hypertension, other vascular disorders (not hypertension), diabetes, corticosteroid use, baseline SDAI score, baseline CDAI score, and number of previous DMARDs.

| 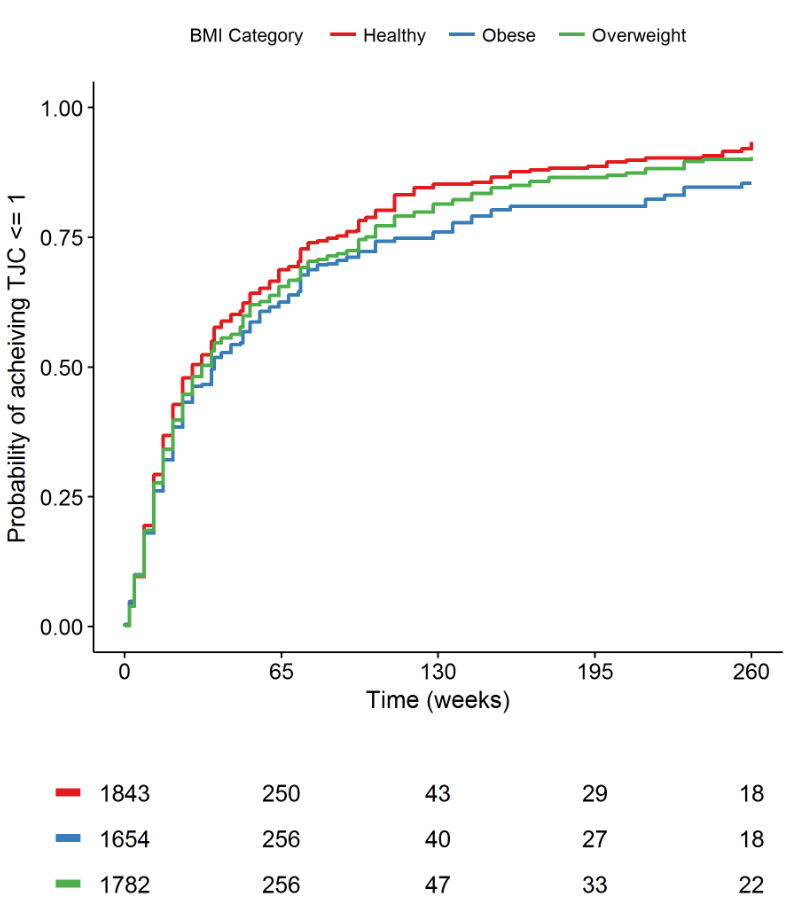 | 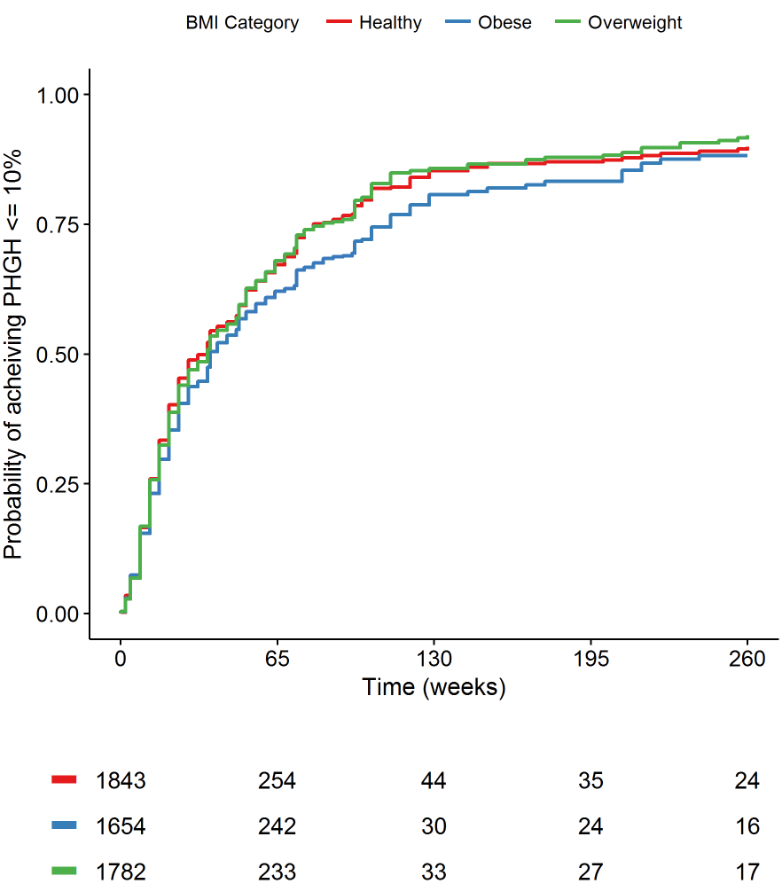 |
| --- | --- |
| 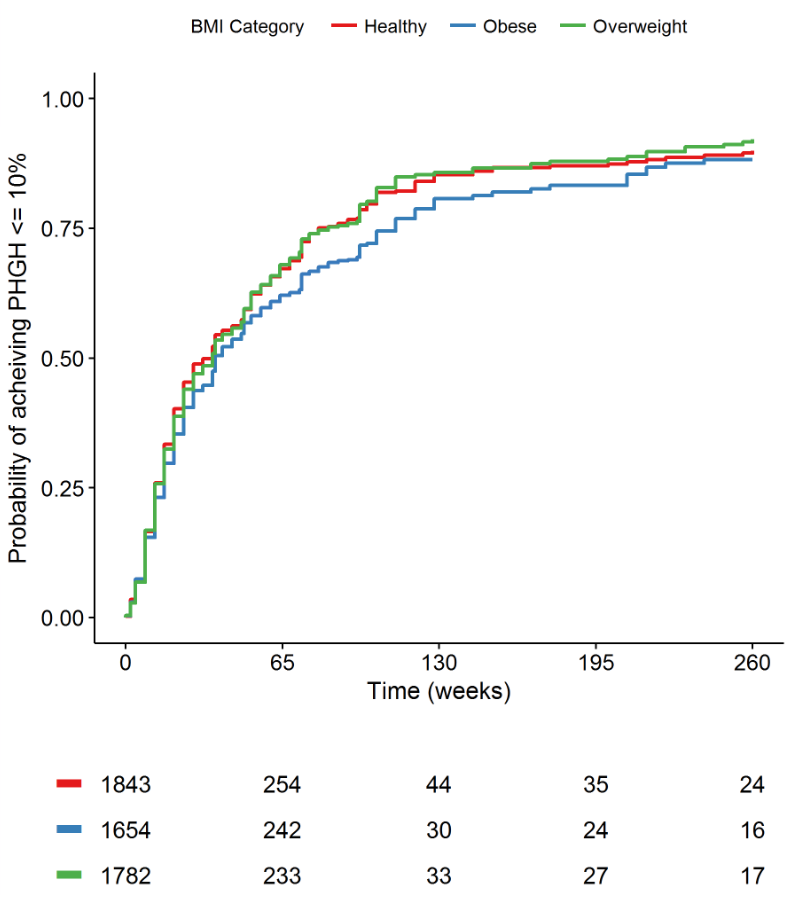 | 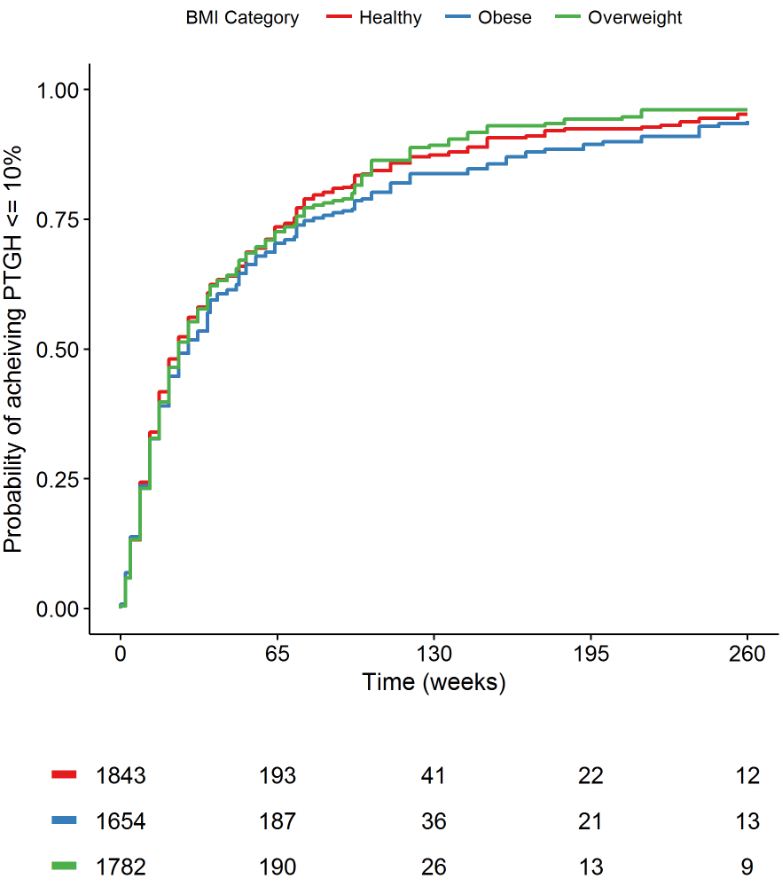 |
| 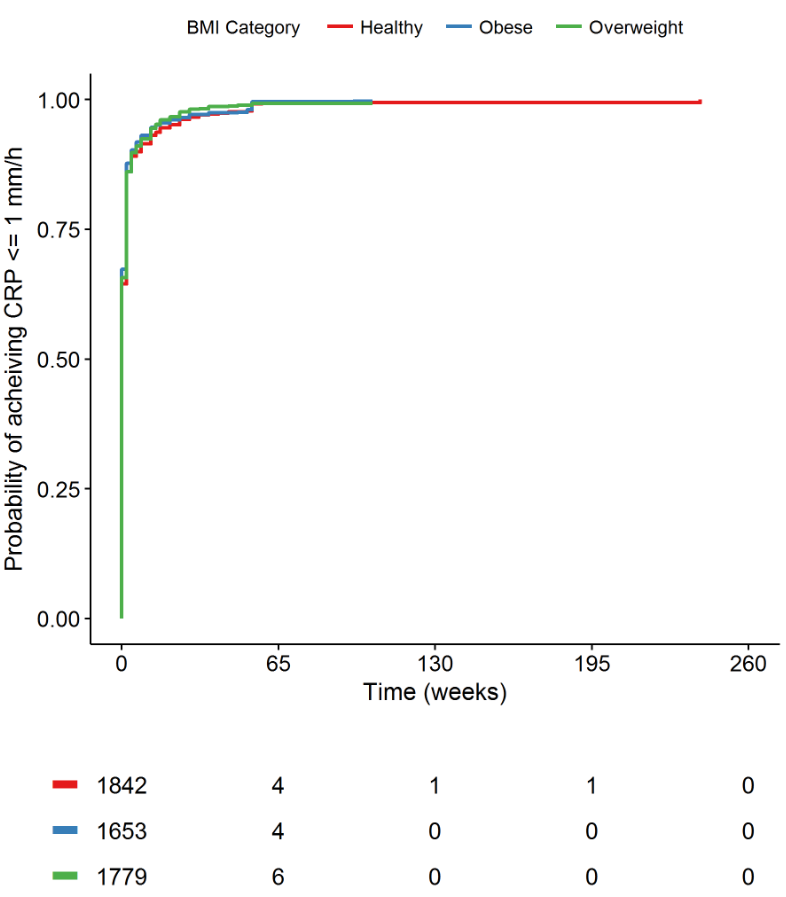 |  |
| Figure S1. Kaplan-Meier estimates of the proportion of rheumatoid arthritis patients achieving remission at least once by BMI category for the individual components of the disease activity measures. The numbers underneath Kaplan- Meier plots indicate the absolute number of patients at risk by time. | |

| 1. **SDAI** | 1. **CDAI** |
| --- | --- |
| 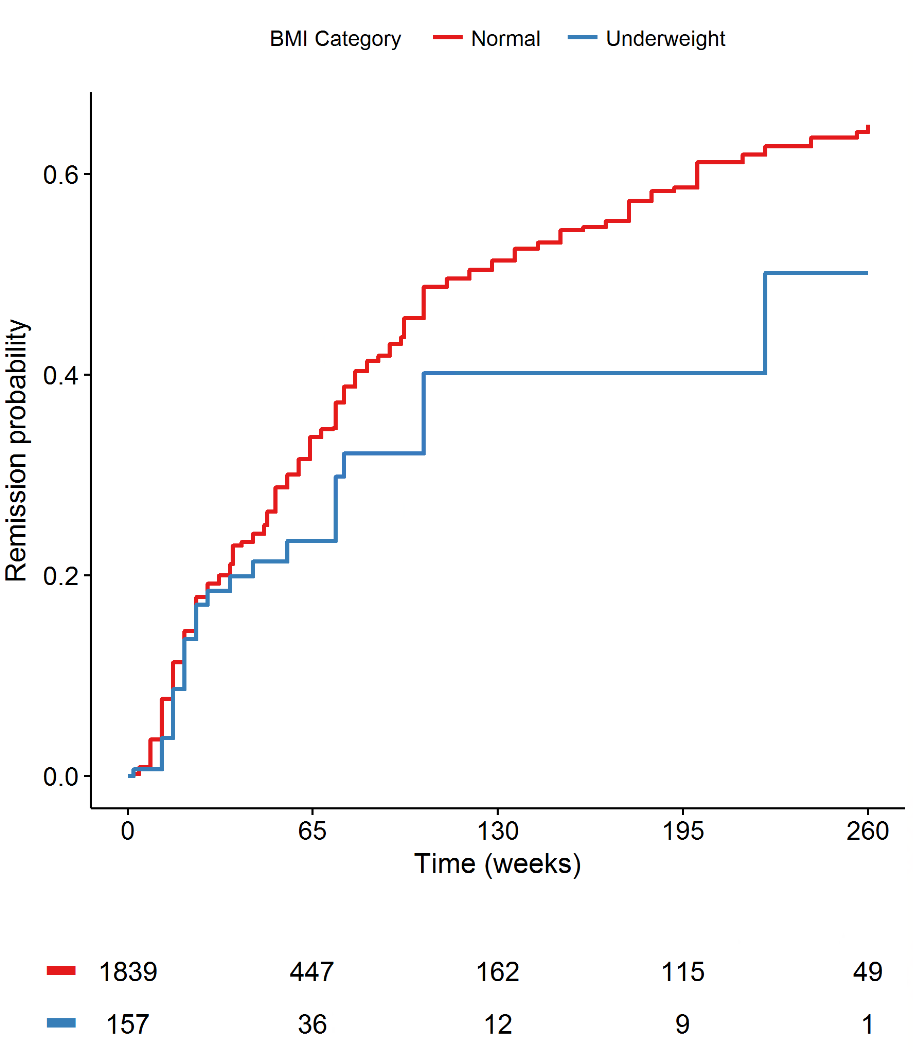 | 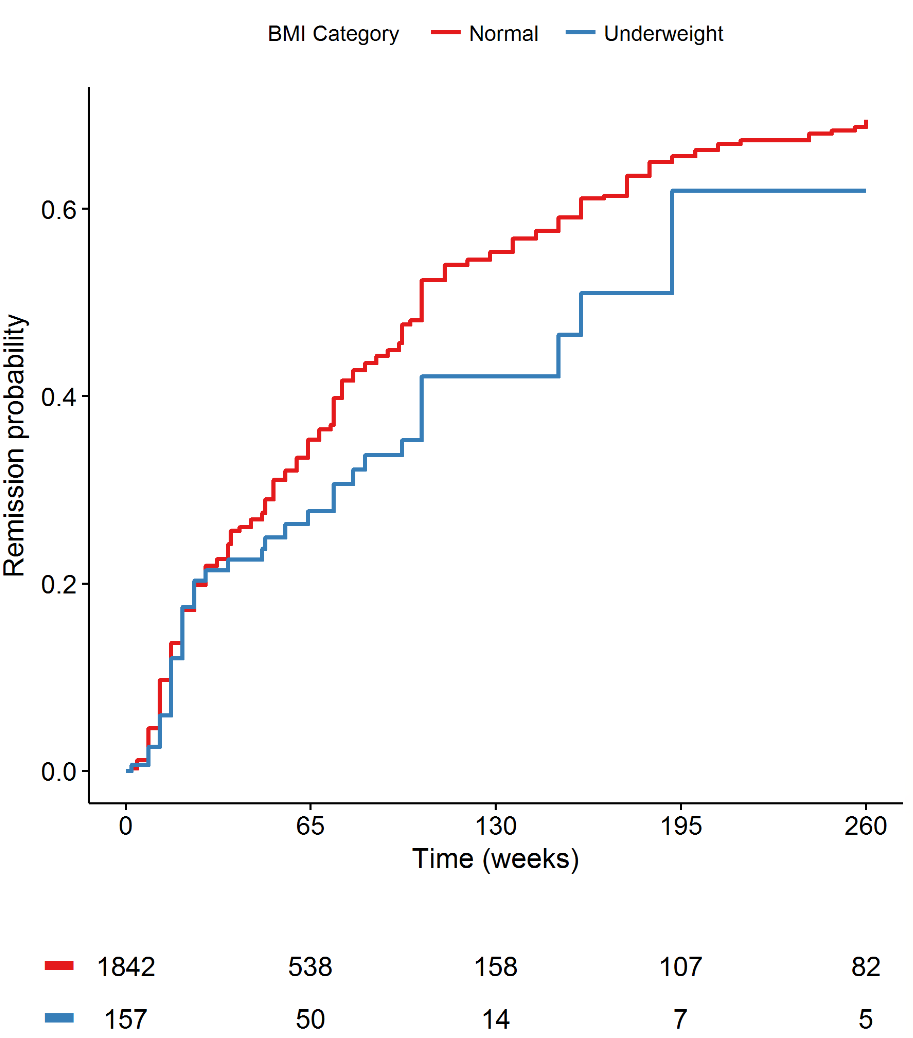 |
| Figure S2. Proportion of underweight rheumatoid arthritis patients achieving remission, at least once, compared to normal weight in the pooled cohort using (A) simplified disease activity index (SDAI) and (B) clinical disease activity index (CDAI) remission. The numbers underneath Kaplan- Meier plots indicate the absolute number of patients at risk by time. | |
